# Supplementary material for: Dual-Functional Peroxidase-Copper Phosphate Hybrid Nanoflowers for Sensitive Detection of Biological Thiols
Source: Int J Mol Sci. 2021 Dec 29;23(1):366. doi: 10.3390/ijms23010366 (PMC8745091; doi:10.3390/ijms23010366)
Supplement: Supplementary file 1 [file ijms-23-00366-s001.zip › ijms-1502455-supplementary.pdf]

International Journal of
Molecular Sciences

Supplementary Materials

Dual-Functional Peroxidase-Copper Phosphate Hybrid Nanoflowers for Sensitive Detection of Biological Thiols

Xuan Ai Le† , Thao Nguyen Le† and Moon Il Kim*

Department of BioNano Technology, Gachon University, 1342 Seongnamdae-ro, Sujeong-gu, Seongnam 13120, Gyeonggi, Republic of Korea; xuanai.le6667@gmail.com (X.A.L); thaonguyen65949@gmail.com (T.N.L)
* Correspondence: moonil@gachon.ac.kr (M.I.K); Tel.: +82-31-750-8563

† These authors contributed equally to this work.

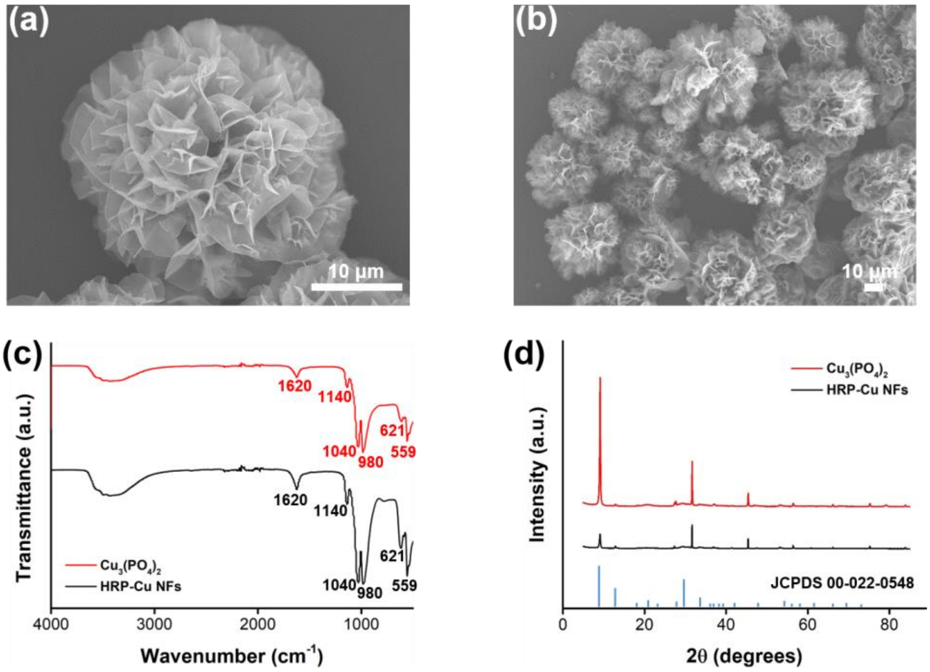

Figure S1. SEM images with a) high magnification and b) low magnification, c) FT-IR spectra, and d) XRD patterns of HRP-Cu NFs.

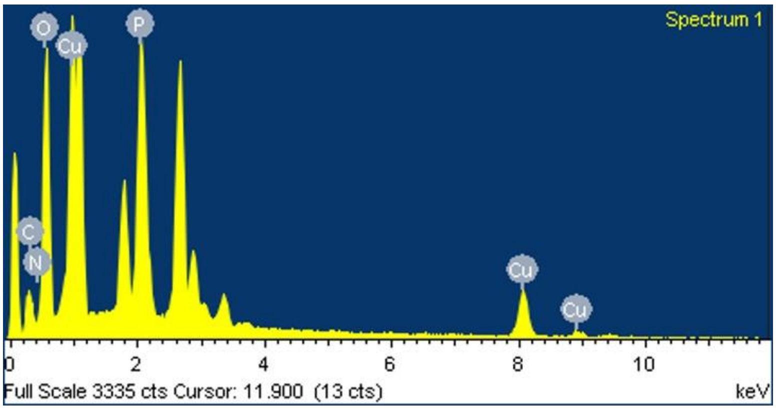

Figure S2. EDS analysis of HRP-Cu NFs.

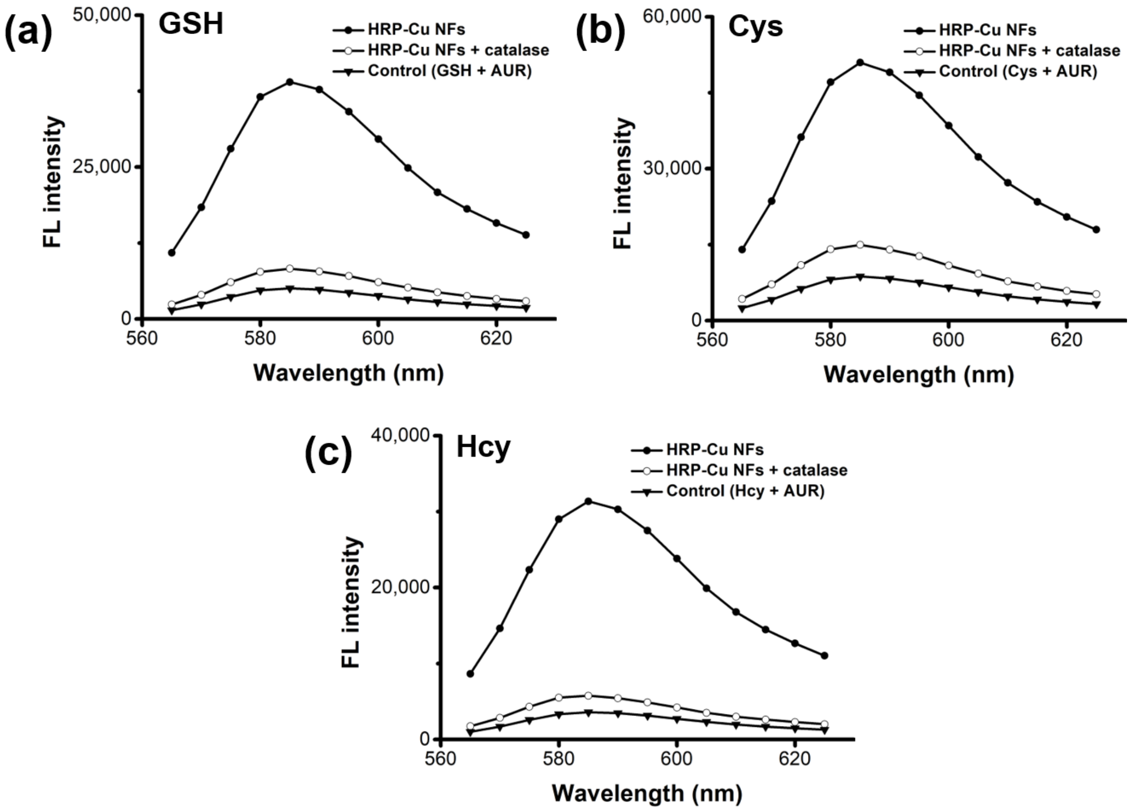

Figure S3. Demonstration for H_2O_2 generation from HRP-Cu NFs-mediated biothiol oxidation via the addition of catalase.

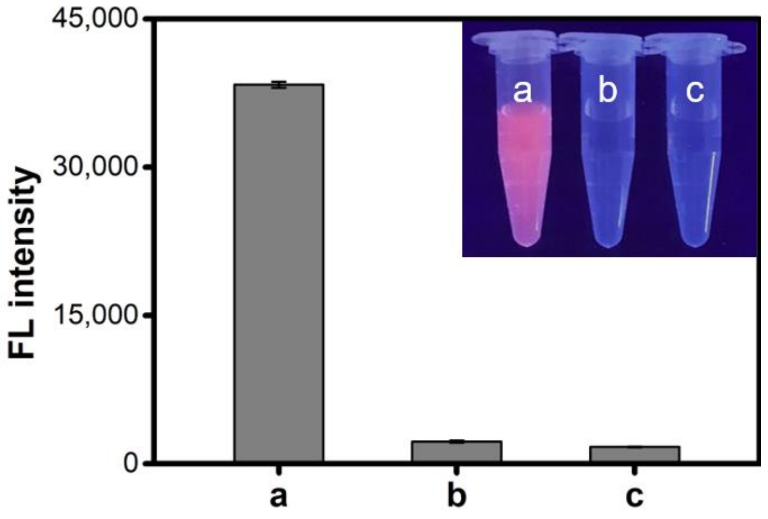

Figure S4. Fluorescence intensities from AUR oxidation in the presence of GSH catalyzed by a) HRP-Cu NFs, b) supernatant solution separated from HRP-Cu NFs, and c) negative control. The supernatant solution was collected by first incubating HRP-Cu NFs (1 mg/mL) in sodium phosphate buffer (800 μL, 10 mM, pH 7.4) for 15 min, followed by centrifugation (10,000 rpm, 1 min).

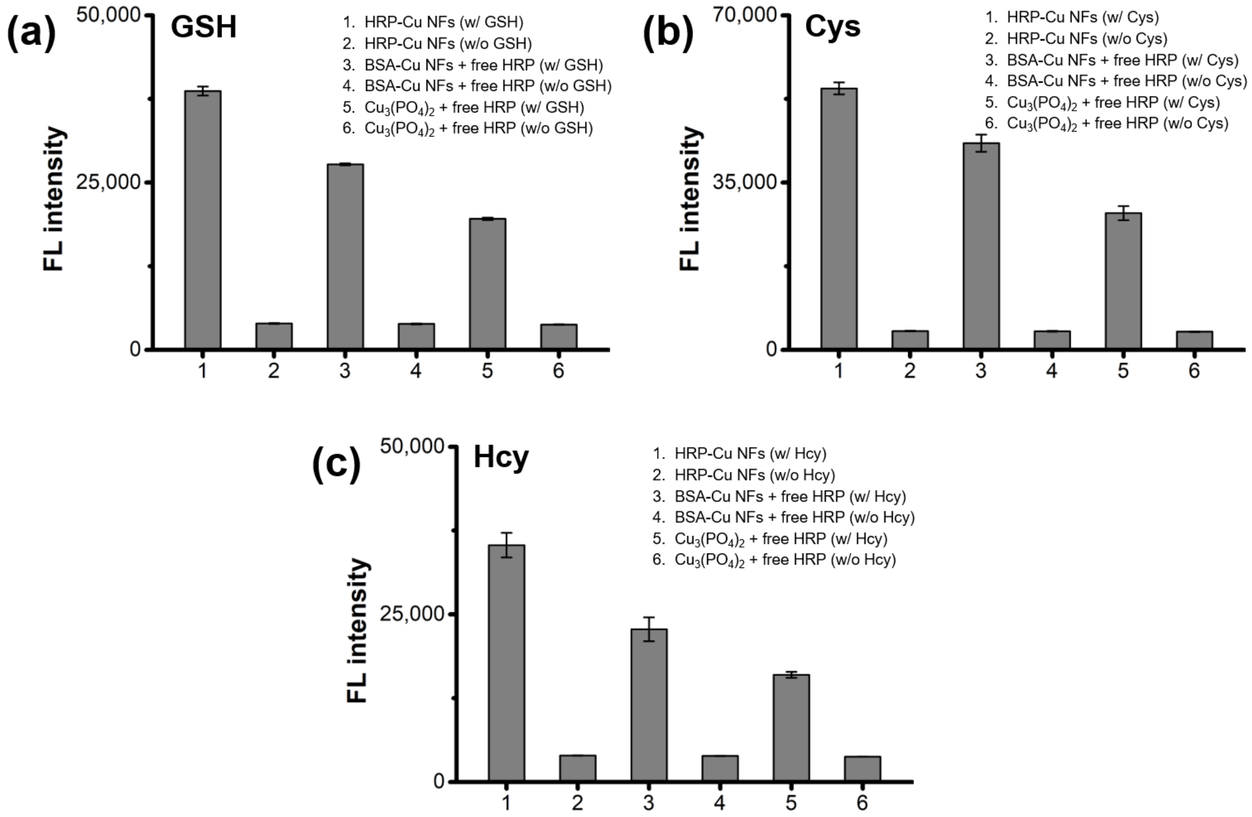

Figure S5. Fluorescence intensities from AUR oxidation promoted by HRP-Cu NFs, BSA-Cu NFs + free HRP, and $Cu_3(PO_4)_2$ precipitates + free HRP in the presence and absence of a) GSH, b) Cys, and c) Hcy.

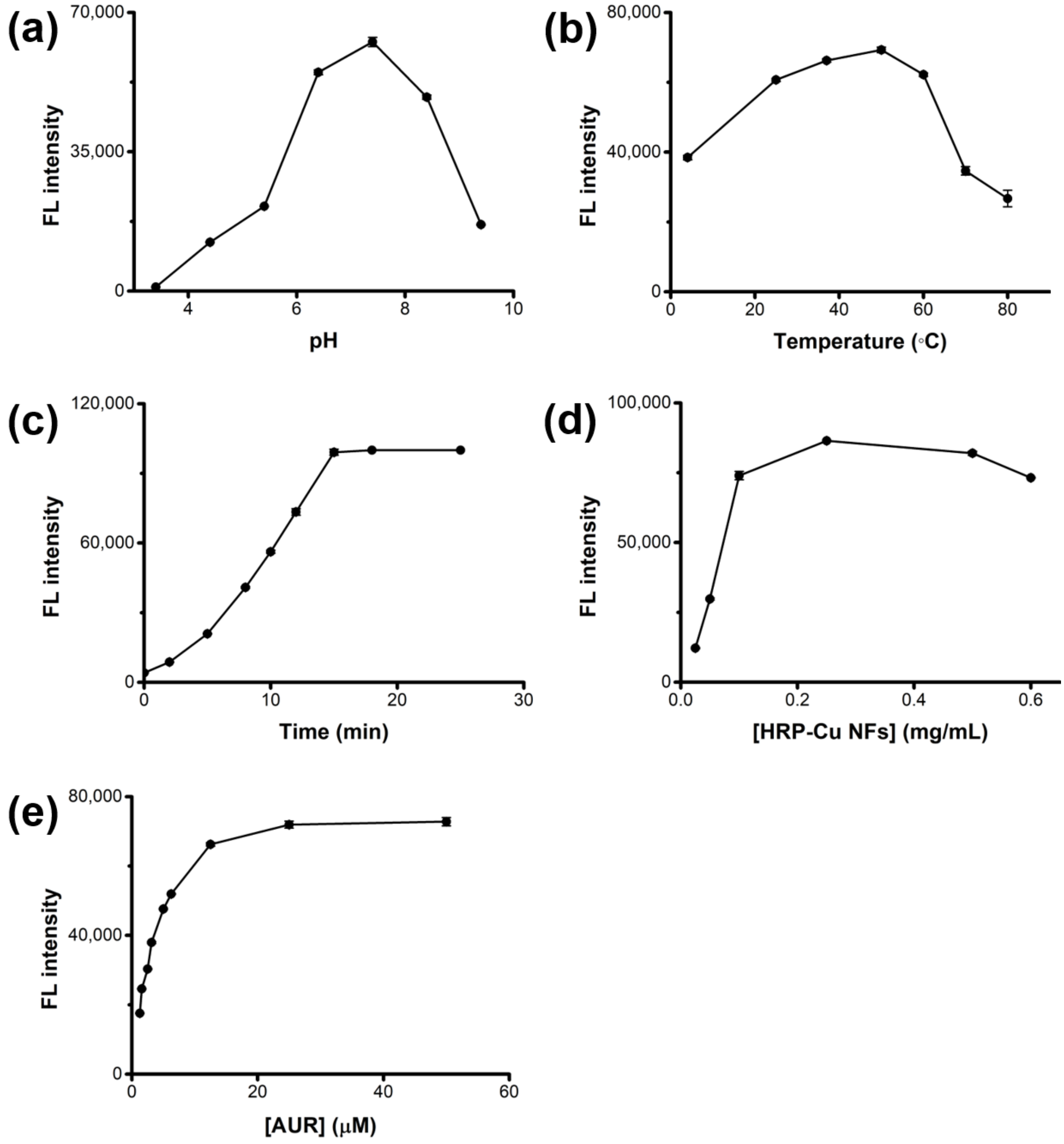

Figure S6. Effects of different reaction conditions on GSH detecting activity of HRP-Cu NFs. a) pH, b) temperature, c) reaction time, and the concentrations of d) HRP-Cu NFs and e) AUR.

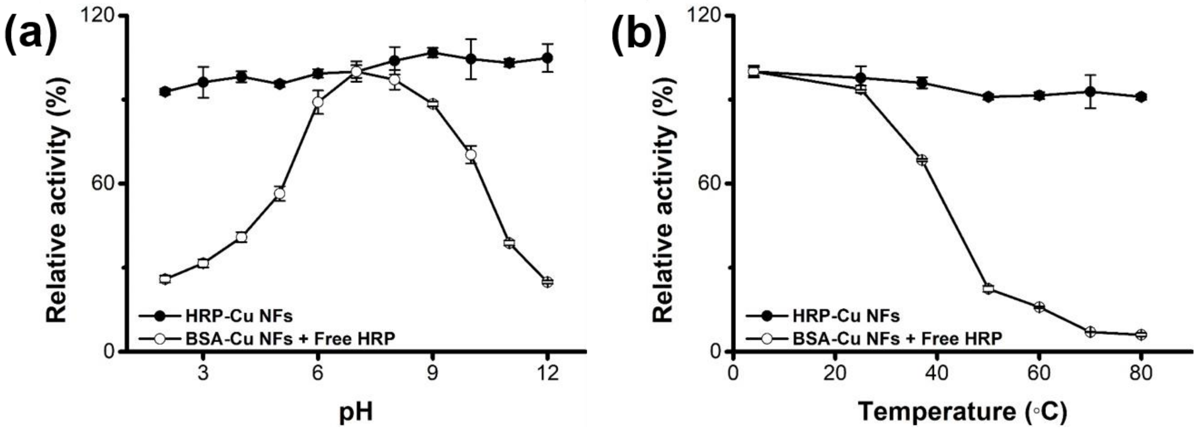

Figure S7. Comparison of a) pH and b) thermal stabilities between HRP-Cu NFs and control system comprising BSA-Cu NFs with free HRP.

Table S1. Comparison of the analytical sensitivities of HRP-Cu NFs-based assay for the determination of GSH, Cys, and Hcy with those of recent reports.

	Method	Linear range (μM)	LOD (μM)	Reference
GSH	Cyclic voltammetry	0.3 – 3.5 × 10^3	0.300	[1]
	Amperometry	857 - 4.1 × 10^3	5.000	[2]
	Colorimetry	1 - 25	0.300	[3]
	Fluorometry	0.1 - 20	0.032	[4]
	Fluorometry	1 - 10	0.300	[5]
	Fluorometry	0.1 - 1.0	13.4 × 10^{-3}	Present study
Cys	Amperometry	5 - 60	1.5	[6]
	Amperometry	5 - 12.6 × 10^3	3.64	[7]
	Colorimetry	0.05 - 10	0.1	[8]
	Colorimetry	1.5 - 6	0.05	[9]
	Fluorometry	0.1 - 100	0.08	[10]
	Fluorometry	1.0 - 110	0.16	[11]
	Fluorometry	0.1 - 1.0	4.5 × 10^{-3}	Present study
Hcy	Amperometry	10 - 80	6.9	[12]
	Amperometry	5 - 200	4.6	[13]
	Colorimetry	0.5 - 3.0	0.5	[14]
	Fluorometry	0 - 25	1.88	[15]
	Fluorometry	0.1 - 1.0	18.3 × 10^{-3}	Present study

Table S2. Determination of GSH in spiked human serum at different dilution factors using HRP-Cu NFs-based assay system.

Dilution factor	Original biothiols (μM)	Added (μM)	Expected (μM)	Measured (μM)	Recovery (%)	CV (%)
1000	0.404	0	0.404	0.397	98.28	3.81
		0.125	0.529	0.533	100.73	2.17
		0.25	0.654	0.639	97.78	1.24
		0.5	0.904	0.862	95.35	1.27
500	0.872	0	0.872	0.834	95.61	2.00
		0.125	0.997	0.913	91.61	0.29
		0.25	1.122	0.938	83.1	1.84
		0.5	1.372	1.116	81.33	5.10

References

1. Narang, J.; Chauhan, N.; Jain, P.; Pundir, C. S. Silver nanoparticles/multiwalled carbon nanotube/polyaniline film for amperometric glutathione biosensor. *Int. J. Biol. Macromol.* **2012**, *50*, 672-678.
2. Yuan, B.; Zeng, X.; Xu, C.; Liu, L.; Ma, Y.; Zhang, D.; Fan, Y. Electrochemical modification of graphene oxide bearing different types of oxygen functional species for the electro-catalytic oxidation of reduced glutathione. *Sens. Actuator B-Chem.* **2013**, *184*, 15-20.
3. Liu, J.; Meng, L.; Fei, Z.; Dyson, P. J.; Jing, X.; Liu, X. MnO_2 nanosheets as an artificial enzyme to mimic oxidase for rapid and sensitive detection of glutathione. *Biosens. Bioelectron.* **2017**, *90*, 69-74.
4. He, L.; Lu, Y.; Gao, X.; Song, P.; Huang, Z.; Liu, S.; Liu, Y. Self-cascade system based on cupric oxide nanoparticles as dual-functional enzyme mimics for ultrasensitive detection of silver ions. *ACS Sustain. Chem. Eng.* **2018**, *6*, 12132-12139.
5. Cai, Q.-Y.; Li, J.; Ge, J.; Zhang, L.; Hu, Y.-L.; Li, Z.-H.; Qu, L.-B. A rapid fluorescence "switch-on" assay for glutathione detection by using carbon dots–MnO_2 nanocomposites. *Biosens. Bioelectron.* **2015**, *72*, 31-36.
6. Silva, C. d. C. C. e.; Breitkreitz, M. C.; Santhiago, M.; Corrêa, C. C.; Kubota, L. T. Construction of a new functional platform by grafting poly(4-vinylpyridine) in multi-walled carbon nanotubes for complexing copper ions aiming the amperometric detection of L-cysteine. *Electrochim. Acta* **2012**, *71*, 150-158.
7. Geng, D.; Li, M.; Bo, X.; Guo, L. Molybdenum nitride/nitrogen-doped multi-walled carbon nanotubes hybrid nanocomposites as novel electrochemical sensor for detection L-cysteine. *Sens. Actuator B-Chem.* **2016**, *237*, 581-590.
8. Lee, J.-S.; Ulmann, P. A.; Han, M. S.; Mirkin, C. A. A DNA−gold nanoparticle-based colorimetric competition assay for the detection of cysteine. *Nano Lett.* **2008**, *8*, 529-533.
9. Chen, S.; Gao, H.; Shen, W.; Lu, C.; Yuan, Q. Colorimetric detection of cysteine using noncrosslinking aggregation of fluorosurfactant-capped silver nanoparticles. *Sens. Actuator B-Chem.* **2014**, *190*, 673-678.
10. Liu, H.; Sun, Y.; Yanga, J.; Hua, Y.; Yanga, R.; Lia, Z.; Qu, L.; Lin, Y. High performance fluorescence biosensing of cysteine in human serum with superior specificity based on carbon dots and cobalt-derived recognition. *Sens. Actuator B-Chem.* **2019**, *280*, 62-68.
11. Dong, W.; Wang, R.; Gong, X.; Liang, W.; Dong, C. A far-red FRET fluorescent probe for ratiometric detection of L-cysteine based on carbon dots and N-acetyl-L-cysteine-capped gold nanoparticles. *Spectroc. Acta Pt. A-Molec. Biomolec. Spectr.* **2019**, *213*, 90-96.
12. Rajaram, R.; Mathiyarasu, J. An electrochemical sensor for homocysteine detection using gold nanoparticle incorporated reduced graphene oxide. *Colloids Surf. B* **2018**, *170*, 109-114.
13. Lawrence, N. S.; Deo, R. P.; Wang, J. Detection of homocysteine at carbon nanotube paste electrodes. *Talanta* **2004,** *63*, 443-449.
14. McKeague, M.; Foster, A.; Miguel, Y.; Giamberardino, A.; Verdin, C. e.; Chan, J. Y. S.; DeRosa, M. C. Development of a DNA aptamer for direct and selective homocysteine detection in human serum. *RSC Adv.* **2013,** *3*, 24415-24422.

15. Barve, A.; Lowry, M.; Escobedo, J. O.; Thainashmuthu, J.; Strongin, R. M. Fluorescein tri-aldehyde promotes the selective detection of homocysteine. *J. Fluoresc.* **2016,** *26*, 731-737.

Copy right: © 2021 by the authors. Submitted for possible open access publication under the terms and conditions of the Creative Commons Attribution (CC BY) license (http://creativecommons.org/licenses/by/4.0/).
